# Health education to promote knowledge about sickle cell disease and newborn screening in pregnant women: a community-based pilot study using the healthy beginning initiative

**DOI:** 10.1186/s12884-024-06498-9

**Published:** 2024-04-26

**Authors:** Osita U. Ezenwosu, John O. Olawepo, Lorraine J. Lacroix-Willliamson, Ijeoma U. Itanyi, Amaka Ogidi, Tonia C. Onyeka, Madeline Gully, Maisha Gregory, Janis L. Breeze, Stephanie Ibemere, Ngozi Idemili-Aronu, Beth E. Molnar, Echezona E. Ezeanolue

**Affiliations:** 1 Center for Translation and Implementation Research, University of Nigeria, Enugu, Nigeria; 2 Department of Pediatrics, College of Medicine, University of Nigeria, Enugu, Nigeria; 3 Department of Health Sciences, Bouvé College of Health Sciences, Northeastern University, Boston, MA USA; 4 Institute for Health Equity and Social Justice Research, Northeastern University, Boston, MA USA; 5 Department of Community Medicine, University of Nigeria, Enugu, Nigeria; 6 Department of Anesthesia/Pain & Palliative Care Unit, University of Nigeria, Enugu, Nigeria; 7 School of Nursing, Bouvé College of Health Sciences, Northeastern University, Boston, USA; 8 Tufts Clinical and Translational Science Institute, Institute for Clinical Research and Health Policy Studies, Tufts University, Tufts Medical Center, Boston, MA USA; 9 Duke University School of Nursing, Duke Global Health Institute, Durham, NC USA; 10 Healthy Sunrise Foundation, Las Vegas, NV USA

**Keywords:** Sickle cell disease, Newborn screening, Health education, Pregnant women, Community-based intervention, Healthy beginning Initiative

## Abstract

**Background:**

Pregnancy presents a critical period for any maternal and child health intervention that may impact the health of the newborn. With low antenatal care attendance by pregnant women in health facilities in Nigeria, community-based programs could enable increased reach for health education about sickle cell disease (SCD) and newborn screening (NBS) among pregnant women. This pilot study aimed to assess the effect of education on the knowledge about SCD and NBS among pregnant women using the Healthy Beginning Initiative, a community-based framework.

**Methods:**

A pre-post study design was used to evaluate knowledge of SCD and NBS in a convenience sample of 89 consenting pregnant women from three communities. Participants were given surveys prior to and following completion of a health education session. McNemar’s test was used to compare the proportion of participants with correct responses. The level of significance was taken as *p <* 0.05.

**Results:**

Compared to pre-test values, post-test values showed that participants understood that SCD is hereditary (93.3% vs. 69.7%), both parents must have at least one gene for someone to have SCD (98.9% vs. 77.5) and blood test is the right way to know if one has SCD (98.8% vs. 78.7%). Also, a large proportion of participants (post-test ~ 89.9%; compared to pre-test ~ 23.6%) understood that the chance of conceiving a child with SCD was 25% for a couple with the sickle cell trait (SCT). Knowledge of the possibility of diagnosing SCD shortly after birth was highly increased in the post test phase of the study when compared to the pre-test phase (93.3% vs. 43.9%, respectively). Concerning the overall knowledge scores, those with high level of knowledge significantly increase from 12.6% pretest to 87.4% posttest (*p* = 0.015).

**Conclusion:**

The health education intervention was associated with significant improvement on almost all measures of SCD knowledge. Focused health education for pregnant women using community structures can improve knowledge of SCD and NBS.

## Background

Sickle cell disease (SCD) is a common inherited blood condition caused by a mutation in the synthesis of hemoglobin (Hb) resulting in either a homozygous or heterozygous state with another abnormal hemoglobin [1]. The most common and most severe form of SCD is the homozygous state known as Sickle Cell Anemia (SCA) due to inheritance of hemoglobin S (HbS) from each parent [1]. The inheritance of HbS in heterozygous state with a normal Hb is known as sickle cell trait which is not part of SCD [1]. 

Worldwide, it is estimated that about 305,000 babies are born annually with SCA [2, 3]. Currently, Nigeria has the highest burden of SCD globally [3] accounting for 50% of new cases in the world [4–6]. Between 50 and 90% of 150,000 Nigerian children born annually with SCD die before their fifth birthday [7, 8] due to late diagnosis and delayed basic prophylactic interventions [9]. The global burden of SCD has been shown to be on the increase due to contributions from Nigeria, India, and Democratic Republic of Congo [3]. The increasing number of SCA will continue to have a major impact on the under-5 mortality rate and particularly on healthcare services and financing in Nigeria [3]. To reduce the global burden of SCD, the role of education to improve the knowledge and awareness of SCD has been emphasized as an important factor [10, 11]. However, controversies exist regarding the appropriate time for education on SCD [12]. 

Some authors suggest this should occur before marriage [13, 14] but others noted that the importance of pre-marital knowledge is invalidated by the observation that some intending couples do not implement the knowledge during marriage [15]. Considering this challenge, some authors have advocated that this should be done during adolescence to provide information early enough to make marital/procreation decisions [16]. A recent study showed an improved effect of health education on adolescents in secondary schools but identified challenges in sustaining the integration of this education into the schools’ curriculum [17]. Other researchers have also noted the importance of education on SCD for youth [18], mothers of under-5 children without SCD [19], and parents of children with SCD [12]. However, despite knowledge at these periods, there still exists a high burden of SCD in Nigeria [3].

Newborn screening (NBS) has been identified as a key strategy for reducing morbidity and mortality associated with SCD [10, 20, 21]. NBS, through early diagnosis and consequent early interventions, is estimated to reduce mortality in approximately 10 million newborns with SCD globally by 2050 [8]. Thus, pregnancy could present an appropriate period for education on SCD and NBS which could impact the demand for NBS. In Nigeria, since only 67% of pregnant women receive any kind of antenatal care from a skilled provider [22], any program aimed at involving pregnant women in a clinic setting will face the challenge of effective reach. Community-based programs could enable increased reach for education on SCD and NBS among the pregnant women [23]. Researchers have identified integration of screening tests into existing community-based programs as a model that is culturally appropriate and acceptable [24, 25]. In a previous study, testing for genotype, hepatitis B, and HIV were successfully integrated into the existing Healthy Beginning Initiative (HBI) – a community-based health care program for pregnant women in congregational settings supported by the President’s Emergency Plan for AIDS Relief (PEPFAR) and National Institutes of Health (NIH) [26–28]. Thus, the HBI provides a culturally adapted platform for screening, linkage and follow up of participants with the aim to respectively identify, treat, and retain them in care [23, 29]. 

Accordingly, this study sought to assess the effect of education on the knowledge about SCD and NBS among pregnant women in their communities.

## Methods

### Participants and setting

This pre-and-post study is part of a larger pilot project to understand the effects of community- and incentive-based approaches on the demand for NBS for SCD in Nigeria. Pregnant women from three local churches were recruited by active church health teams (CHTs) in three respective communities in Enugu State, Southeast Nigeria. Enugu State has an estimated population of 3.2 million people [30]. In 2013, the HBI was deployed in 40 communities in Enugu, where our team has a history of working with religious leaders [23, 26]. HBI is a community-based framework for maternal and child health which consists of three strategic components involving prayer session, baby shower and baby reception with integration of health education, nutritional counselling and tests which offer the mother-child dyad with a healthy start [26, 29]. 

### Sample size and eligibility criteria

Sample size was limited by the budget constraints for this pilot project. A convenience sample of 90 pregnant women who indicated interest in the HBI were invited to participate in this study. The inclusion criteria were women aged 15–49 years in their second or third trimester of pregnancy who attended the selected churches [31], and had working contact phone numbers.

### SCD and NBS education guide

The team adapted questionnaire for SCD and NBS education from a number of previous studies [16, 19]. The SCD and NBS Education Guide included:

1. An overview of SCD – (a) What is SCD? (b) How common is SCD? (c) What are the differences between SCD and Sickle Cell Trait? (d) How babies get SCD and inheritance patterns.
2. A summary of SCD screening – (a) Why is it important to screen pregnant women for SCD? (b) How are people screened or diagnosed for SCD?
3. A summary of NBS for SCD – (a) What is NBS? (b) How soon after birth should NBS be conducted? (c) Where and how is NBS performed? (d) What does the result of the NBS mean? (e) Why is NBS for SCD important? (f) What should a mother do if her child is diagnosed with SCD?

### Study procedures

The study protocol was explained to the clergy and the Church Health Team (CHT) in each church. The CHTs were subsequently trained by the study team on how to conduct a baby shower, a health education and entertainment session to celebrate pregnancy [28, 32]. The research team also trained the CHTs on basic genetic counseling and local resources for NBS. Baby showers were conducted at the three churches to screen the ninety pregnant women. On the day of baby showers, the CHTs followed the procedures of the HBI framework [29]. HBI is a culturally adapted approach that relied on the wide distribution of churches and church-based community networks to address barriers to screening for pregnant women by providing education and counseling (knowledge), on-site, free testing [33]. It was designed to reduce barriers to screening including knowledge, access and stigma while its culturally appropriate baby showers is aimed at reducing losses to follow-up and increase linkage to care at post-delivery period [26]. 

Briefly, during worship, the priest invited all the pregnant women and their male partners for prayers for healthy pregnancy and safe delivery. The priest provided information on HBI and notified the pregnant women that the baby shower will be held during the week. All the pregnant women who indicated interest were given information about the program and their questions answered by the CHT. The CHTs then confirmed eligibility for the study by asking the qualifying questions in the inclusion criteria. The study was explained to the potential participants in English and Igbo (the local Nigerian language). Written informed consent was obtained from those willing to participate in the study prior to completing the study survey forms.

On the day of the baby shower, the CHTs convened the pregnant women for the program. Using the education guide, educational sessions at each of the three churches were delivered by a team led by either a hematologist or a program manager. All team leads have experience in running over 100 baby showers for funded research studies between 2013 and 2019 [27, 28, 31, 32]. The education session with questions and answers lasted approximately 30–45 min. In addition, during the baby showers the CHTs took basic health measurements including height, weight, and blood pressure. Hb genotype screening was conducted using HemoTypeSC™ rapid test with on-the-spot results delivered to the participants. The HemoTypeSC test has been tested and validated in Nigeria and sub-Saharan Africa, showing a sensitivity of 93.4% and specificity of 99.9% compared to the gold standard test – high performance liquid chromatography (HPLC) [34]. While presenting the confidential test results to the participants, the CHT also provided them with basic post-test counseling and referral for NBS.

### Data collection

Each participant was given an investigator-assisted pre-health-education survey (pre-test) which included a mix of true/false, multiple choice, and open-ended questions. Respondents were asked demographic information (level of education, occupation, and religion) and SCD-specific information such as awareness of genotype, (example: *Do you know your Hb Genotype? )*, time of awareness (*When did you first know your genotype?*), and perception of sickle cell trait (SCT; *People who have sickle cell trait have symptoms of sickle cell disease*), SCD (*How does someone get Sickle Cell Disease?*), and NBS (*How soon after delivery should a baby get screening for sickle cell disease?*). Upon completion of the health education session and the health tests, participants completed a post-test, which included the same questions as the pre-test, to assess changes in SCD and NBS knowledge.

Six questions were summed to generate the total SCD Knowledge Score:

1. *How does someone get Sickle Cell Disease? (multiple options provided)*
2. *For someone to get sickle cell disease, both parents must have at least one sickle cell disease gene. (T / F)*
3. *How would someone know if they have sickle cell disease? (multiple options provided)*
4. *It is important to know your Hb genotype even if you don’t have any symptoms. (T / F)*
5. *People who have sickle cell traits have symptoms of sickle cell disease. (T / F)*
6. *If two people with sickle cell traits marry, the chance that their child will have sickle cell disease is ? (Multiple options provided)*

### Data analysis

Statistical analysis for this study was performed using SAS software, version 9.4 of the SAS System for Windows. McNemar’s tests were used to compare frequencies of correct responses, and differences in total scores pre-test versus post-test. The level of significance was taken as *p* < 0.05. Items with multiple response choices were recoded into binary correct vs. incorrect response, to generate chi-square test statistic for the paired data. To generate the SCD knowledge score, the six test questions were summed with 1 point assigned for a correct answer, and a 0 for an incorrect answer. Total scores of ≤ 2 was graded as low SCD knowledge, scores 3–4 were intermediate SCD knowledge, and scores ≥ 5 graded as high SCD knowledge.

### Ethical considerations

This study was approved by the Institutional Review Board of University of Nigeria Teaching Hospital, Enugu, Nigeria (IRB Number – UNTH/HREC/2021/11/284) and Northeastern University Boston, Massachusetts (IRB #: 22-03-38).

## Results

### Description of study participants

A total of 89 pregnant women were enrolled between March and May 2022 across the three communities in Enugu State, South-East, Nigeria. Table 1 details the characteristics of the study population. Participant ages ranged between 15 and 45 years, with a mean and standard deviation of 28.2 ± 6.47 years. The majority (95.5%, *n* = 85) were married and had at least one child (84.3%, *n* = 74). Fifty-six participants (62.9%) completed senior secondary school, and almost half were employed as some type of trader (44.9%, *n* = 40). Prior to the health education, most participants had heard of SCD (92.1%, *n* = 82), and slightly more than half had learned about it in their communities (58.5%, *n* = 48) versus a clinical setting (40.3%, *n* = 33). Among women who were aware of their genotype (75.3%, *n* = 67), most knew their genotype before marriage or during marriage preparation (66.3%, *n* = 59) (Table 2).

**Table 1:** Baseline description of study participants

*Sample Characteristics* (*N* = *89)*		
	N	%
Demographic Information		
Age, years		
15–19	7	7.9
20–29	52	58.4
30–40	24	27.0
40–45	6	6.7
Community Site		
Agbogugu	30	33.7
Awgu	30	33.7
Onoli	29	32.6
Marital Status		
Married	85	95.5
Single	4	4.5
Highest Level of Education		
Primary school	6	6.8
Junior secondary school	18	20.2
Senior secondary school	56	62.9
Post-secondary	9	10.1
Type of Employment		
Applicant	8	9.0
Civil Servant	4	4.5
Farmer	24	27.0
Trader	40	44.9
Other	13	14.6
Number of Children		
None, first pregnancy	14	15.7
1	20	22.5
2	26	29.2
3	12	13.5
4	12	13.5
5	3	3.4
6	2	2.2
Genotype		
AA	67	75.3
AS	21	23.6
SS	1	1.1

**Table 2:** Awareness of genotype

	N	%
Have you heard about sickle cell disease?		
Yes	82	92.1
No	7	7.9
If yes, from where?		
Clinic	33	40.3
Community setting	48	58.5
Missing	1	1.2
Do you know your genotype?		
Yes No	67 22	75.3 24.7
When did you first know your genotype?		
Before marriage / During marriage preparation	59	66.3
During first pregnancy	6	6.7
During other pregnancies	1	1.1
Cannot recall	7	7.9
While sick in the hospital	1	1.1
Missing	15	16.9

### Sickle cell disease (SCD) knowledge

The results show that health education had a statistically significant effect (at the *p* < 0.05 level) on all but one measure of SCD knowledge (Table 3). When compared to pre-test values, post-test results showed participants understood that SCD is hereditary and is a condition that someone is born with (69.7% vs. 93.3%, *p* < 0.0001), and that for someone to get SCD, both parents must have at least one gene (77.5 vs. 98.9%, *p* < 0.0001). Post-test, all women understood, that it was important to know their Hb genotype (100%, *n* = 89) as compared to knowledge in the pre-test phase (85.4%, *n* = 76), and nearly all participants understood in the post-test phase, that a blood test would be the way to know if they had SCD as compared to pre-test phase (98.9% vs. 78.7%, *p* < 0.0001).

**Table 3:** Health education and knowledge of sickle cell disease among pregnant women *N* = 89

	Before health education n (%)	After health education n (%)	*p*-value	*p*-value sig. level
How does someone get Sickle Cell Disease?				
You are born with it (hereditary)	62 (69.7)	83 (93.3)	< 0.0001	***
You get it from a blood transfusion	6 (6.7)	6 (6.7)		
Don’t know	19 (21.4)	0 (0.0)		
Other ways	2 (2.2)	0 (0.0)		
For someone to get sickle cell disease, both parents must have at least one sickle cell disease gene				
True	69 (77.5)	88 (98.9)	< 0.0001	***
False	3 (3.4)	1 (1.1)		
Don’t know	17 (19.1)	0 (0.0)		
How would someone know if they have sickle cell disease?				
Get their blood tested	70 (78.7)	88 (98.9)	< 0.0001	***
Get their urine tested	1 (1.1)	0 (0.0)		
Don’t know	18 (20.2)	0 (0.0)		
Missing	0 (0.0)	1 (1.1)		
It is important to know your Hb genotype even if you don’t have any symptoms.				
True	76 (85.4)	89 (100.0)	0.0003	**
False	0 (0.0)	0 (0.0)		
Don’t know	13 (14.6)	0 (0.0)		
People who have sickle cell trait have symptoms of sickle cell disease.				
True	48 (54.0)	42 (47.2)	< 0.0001	***
False	18 (20.2)	45 (50.6)		
Don’t know	22 (24.7)	2 (2.2)		
Missing	1 (1.1)	0 (0.0)		
If two people with sickle cell trait marry, the chance that their child will have sickle cell disease is:				
1 in 4 (25%)	21 (23.6)	80 (89.9)	< 0.0001	***
1 in 2 (50%)	10 (11.3)	3 (3.4)		
1 in 1 (100%)	18 (20.2)	2 (2.2)		
Zero chance	0 (0.0)	1 (1.1)		
Don’t know	40 (44.9)	3 (3.4)		
Have you heard of Newborn Screening?				
Yes	30 (33.7)	80 (89.9)	< 0.0001	***
No	59 (66.3)	8 (9.0)		
Missing	0 (0.0)	1 (1.1)		
Are you aware that sickle cell disease can be diagnosed soon after birth?				
Yes	39 (43.8)	83 (93.3)	< 0.0001	***
No	49 (55.1)	6 (6.7)		
Missing	1 (1.1)	0 (0.0)		
How soon after delivery should a baby get screening for sickle cell disease?				
Soon after birth within 6 weeks	39 (43.9)	83 (93.3)	< 0.0001	***
When infant is 6 weeks − 6 months old	2 (2.2)	0 (0.0)		
After 6 months-1 years	0 (0.0)	3 (3.4)		
When child is 2–6 years	3 (3.4)	0 (0.0)		
When child is getting signs and symptoms/ sick	2 (2.2)	1 (1.1)		
Incomplete response (take them to hospital)	0 (0.0)	1 (1.1)		
Immediately after giving him or her blood test	1 (1.1)	0 (0.0)		
Once/ Once year	3 (3.4)	0 (0.0)		
Don’t know	37 (41.6)	0 (0.0)		
Missing	2 (2.2)	1 (1.1)		
If a baby has sickle cell trait, what should the parent do?				
Counseling/ give child proper education/ let child know about it at the appropriate time	20 (22.5)	27 (30.4)	0.052	
Don’t know	27 (30.4)	2 (2.2)		
Don’t ever marry another person with the trait / AS	6 (6.7)	3 (3.4)		
Get the child tested in the hospital / treatment/ medication/ take care of baby	30 (33.7)	33 (37.1)		
Nothing/ leave baby/ no side effect	4 (4.5)	22 (24.7)		
Parent should do test	1 (1.1)	0 (0.0)		
Missing	1 (1.1)	2 (2.2)		
If a baby has sickle cell disease, what should the parent do?				
They should take the baby to hospital for treatment/ follow-up with the treatment/ go to hospital for constant transfusion/ both parents to report to doctor	61 (68.6)	87 (97.8)	< 0.0001	***
Advice the child about the health implications of it	0 (0.0)	1 (1.1)		
They will fight for it/ keep the child/ take care of the child/ leave it to God	3 (3.4)	0 (0.0)		
The parents will go for screening test	1 (1.1)	0 (0.0)		
Don’t know	22 (24.7)	0 (0.0)		
Missing	2 (2.2)	1 (1.1)		

Note. *p-value < 0.05; **p-value < 0.01; *** *p*-value < 0.0001

### Sickle cell trait (SCT) knowledge and awareness

Knowledge regarding the sickle cell trait (SCT) had mixed results, as shown in Table 3. After the health education, a large proportion of pregnant women (89.9%, *n* = 80) understood that with sexual relationship between two individuals (male and female) with SCT, the chance that their offspring will have SCD is 25%, compared to 23.6% (*n* = 21) of the women at pre-test. However, only half of participants in the post-test phase (50.6%, *n* = 45) correctly understood that people with the SCT do not have symptoms of sickle cell disease, even though this was a significant improvement from pre-test (20.2%, *n* = 18). The only measure that did not significantly change after health education was the understanding around what to do if a child was born with the sickle cell trait. Although there was a significant increase in understanding that counseling would be the appropriate action if a baby has SCT post-test as compared to pre-test (30.4% vs. 22.5%, *p* < 0.052), over a third of respondents in the post-test phase of this study (37.1%, *n* = 33) incorrectly stated that the baby needed some type of treatment or medication at a hospital, a response rate similar to the pre-test phase (33.7%, *n* = 30).

### Newborn screening (NBS) knowledge and awareness

Almost all participants were aware that SCD can be diagnosed soon after birth post-health education as compared to pre-health education (93.3% vs. 43.9%, *p* < 0.0001). The same proportion post-test (93.3%, *n* = 83) correctly indicated that testing should occur within 6 weeks of a child’s birth, a significant increase from pre-test (43.9%, *n* = 39). Overwhelmingly, participants understood at post-test that if babies were diagnosed with SCD, that they should take the baby to seek treatment (97.8%, *n* = 87) as compared to their understanding prior to the health education intervention (68.6%, *n* = 61).

### Level of SCD knowledge scores

**Fig. 1:**
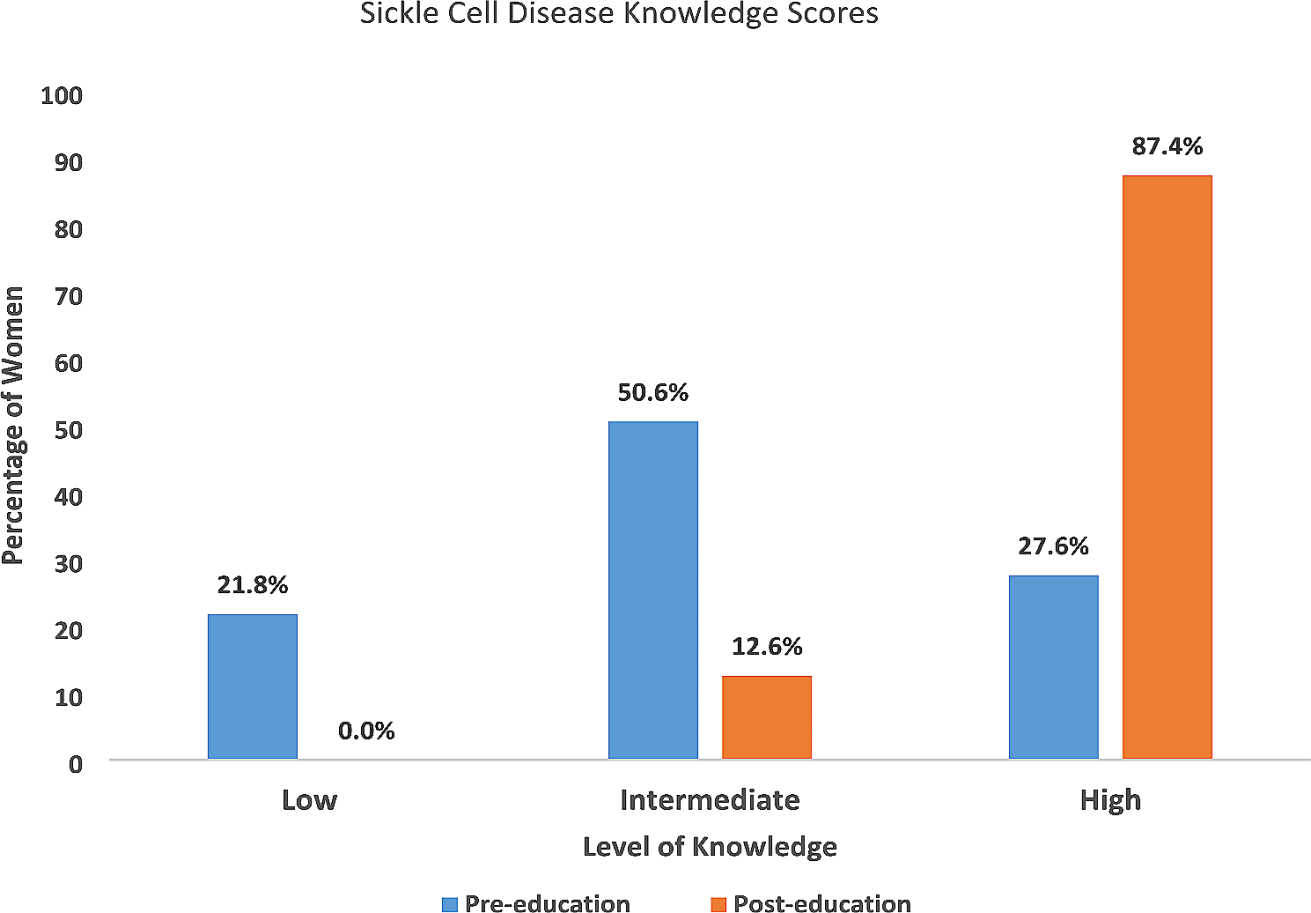
Effect of health education on level of SCD knowledge among pregnant women (*N* = 87)

The frequency distribution of levels of knowledge before and after the health education intervention are displayed in Fig. 1. Two participants did not answer all questions that made up the total sum score, and therefore did not obtain an overall knowledge level score. At post-test, participants had increased their knowledge, so that nobody still had low knowledge while 87.4% had high levels of knowledge which was statistically significant (χ^2^ = 24.83, *p* = 0.0157).

## Discussion

Our study demonstrated that it is feasible to conduct a health education intervention on SCD and NBS among pregnant women within the community setting. It also provided evidence that pregnancy presents an ideal period for health education on SCD and NBS as increased overall level of knowledge and improvement in specific knowledge questions were noted. Health education sessions offered in a community setting may be an effective means to increase knowledge of SCD and NBS [11]. 

The feasibility of conducting intervention programs among pregnant women in the community has been previously demonstrated [31, 32, 35]. The earlier studies integrated HIV testing [31], Hb genotype testing [32], and mental health screening [35] into existing community-based programs. Our study focused on health education in the community setting since such a setting affords the opportunity to increase the reach of educational programs. Unlike other studies on SCD education in non-community settings which focused on the adolescents [17], youth [18], mothers of children with SCD [12] and mothers of under-five children [19], ours involved pregnant women. Education at such period has the potential of increasing the demand for NBS leading to the identification of newborns with SCD for early intervention and consequent reduction in morbidity and mortality associated with SCD [32]. The improvement in knowledge of SCD and NBS following education in this study was in keeping with reports from other studies [11, 17, 36, 37]. The participants’ improved knowledge of SCD may have been remotely affected by their earlier knowledge of their genotype.

There is still limited understanding of the difference between SCT and SCD. This is similar to the experience in previous studies [12, 38] by our research team where, respectively, a significant proportion of parents of children with SCD thought that SCT was a mild form of SCD [12] and majority of adolescents incorrectly thought that people with SCT have the symptoms of SCA [38]. Participants in one of the previous studies [12] suggested health education via community engagements as a potential method to improve understanding [12]. Other authors have identified increased frequency of health education campaigns as a modified strategy to increase the understanding of SCT and SCD [36]. In our study, the participants did not adequately comprehend what they should do if their infants had SCT highlighting the need for more education. Frequent education of these mothers could prepare them to provide guidance to their children as they mature into adolescents and young adults. Guiding young people on marital decisions and discussion on choosing the right partner have been shown as a form of primary prevention of SCD [39]. 

### Strength of the study

This study draws its strength from its community-based nature. Since most pregnant women in Nigeria do not attend care in the hospital facilities [22, 40], a program that takes care to them within their communities will most likely improve accessibility and reach. Even among women that have attended antenatal care, the education at the community level provides opportunity to consolidate their knowledge about SCD and NBS. To the best of our knowledge, this is the first study to educate pregnant women on NBS for SCD using a well-structured evidence-based community intervention like the HBI [29]. 

### Limitation of the study

A major limitation of this study was the sample size which was restricted by the small budget available. A larger sample size will improve the generalizability of the findings of this study. Again, the health education lasted little below an hour and a further study could improve on this.

### Policy, clinical and research implications of our findings

The feasibility of conducting this health education among pregnant women in a community setting shows that governments could sponsor larger studies to provide further evidence. The success of this health education intervention has the potential to reduce the burden of SCD in Nigeria. There may be need for the Nigerian government and governments in other African nations affected by SCD to develop a policy framework that will build partnerships with community settings and places of worship to serve as a platform for the dissemination of SCD education services to pregnant women and others.

## Conclusion

This pilot study demonstrates that focused health education among pregnant women has a positive effect on their knowledge of SCD and NBS. Regular conduct of such educational programs has the potential to dispel any cultural myths and practices surrounding SCD and NBS. Results from this pilot study will ultimately guide the development of a large-scale community-based intervention to address the research and program gaps for SCD newborn screening in Nigeria.

## Data Availability

The data that support the findings of this study are available from the first and corresponding author, Osita Ezenwosu.

## Abbreviations

CHTs: Church Health Teams
HBI: Healthy Beginning Initiative
Hb: Hemoglobin
HbS: Hemoglobin S
HPLC: High Performance Liquid Chromatography
NBS: Newborn Screening
SCA: Sickle Cell Anemia
SCD: Sickle Cell Disease
SCT: Sickle Cell Trait
